# Chemical staining of particulate organic matter for improved contrast in soil X-ray µCT images

**DOI:** 10.1038/s41598-020-79681-5

**Published:** 2021-01-11

**Authors:** Peter Maenhout, Stefaan De Neve, Joanna Wragg, Barry Rawlins, Jan De Pue, Luc Van Hoorebeke, Veerle Cnudde, Steven Sleutel

**Affiliations:** 1 Research Group of Soil Fertility and Nutrient Management, Department of Environment, Ghent University, Coupure Links 653, 9000 Gent, Belgium; 2 British Geological Survey, Keyworth, Nottingham, NG12 5GG UK; 3 Research Group of Soil Physics, Department of Environment, Ghent University, Coupure Links 653, 9000 Gent, Belgium; 4 Radiation Physics Research Group-UGCT, Department of Physics and Astronomy, Ghent University, Proeftuinstraat 86, 9000 Gent, Belgium; 5 PProGRess-UGCT, Department of Geology, Ghent University, Krijgslaan 281, 9000 Ghent, Belgium; 6 Department of Earth Sciences, Utrecht University, Princetonlaan 8a, 3584 CB Utrecht, The Netherlands

**Keywords:** Carbon cycle, Carbon cycle, Carbon cycle

## Abstract

Degradability of organic matter (OM) in soil depends on its spatial location in the soil matrix. A recent breakthrough in 3D-localization of OM combined dual-energy X-ray CT-scanning with OsO_4_ staining of OM. The necessity for synchrotron-based µCT and the use of highly toxic OsO_4_ severely limit applications in soil biological experiments. Here, we evaluated the potential of alternative staining agents (silver nitrate, phosphomolybdenic acid (PMA), lead nitrate, lead acetate) to selectively enhance X-ray attenuation and contrast of OM in CT volumes of soils containing specific mineral soil particle fractions, obtained via lab-based X-ray µCT. In comparison with OsO_4_, administration of Ag^+^ and Pb^2+^ resulted in insufficient contrast enhancement of OM versus fine silt (< 20 µm) or clay (< 2 µm) mineral particles. The perfusion procedure used in this work induced changes in soil structure. In contrast, PMA staining resulted in a selective increase of OM’s attenuation contrast, which was comparable to OsO_4_. However, OM discrimination from other soil phases remained a challenge. Further development of segmentation algorithms accounting for grey value patterns and shape of stained particulate OM may enable its automated identification. If successful in undisturbed soils, PMA staining may form an alternative to OsO_4_ in non-synchrotron based POM detection.

## Introduction

Soil architecture (structure) and organic matter (OM) turnover in soil are related in a number of ways^1–6^. In temperate soils OM is often the major driver of aggregate formation and stabilization^7^ as a direct binding agent or as an energy source for micro-organisms, subsequently forming microbial decomposition products. The metabolites (e.g. glycoproteins, polysaccharides) contained in the microbial mucilage act as binding agents. These contribute to soil structure stabilization, due the affinity of their inherent functional groups for mineral surfaces^3,8–10^.

Physical inaccessibility (physical protection) within aggregates protects OM from degradation. The compartmentalization of OM, microbial biomass and microbial grazers along with reduced O_2_ diffusion into aggregates^11–15^ result in environmental conditions within aggregates that protect organic matter. Disturbance of these protective environments, via e.g. changes in land use management, can result in significant losses of specific soil organic matter (SOM) pools^16^. In particular, particulate organic matter (POM) which mainly consists of non-degraded plant and animal residues, is most often considered a very labile SOM fraction^17^ and an important nutrient source for microorganisms^18^ when not occluded in fine aggregates. Globally, POM may represent 75–230 Pg of soil carbon that is highly sensitive to degradation if not located in physically protected soil environments. The stabilization of plant residues via fragmentation in POM^19^ is significant for C sequestration^20^. A spatially explicit approach is needed to unravel the underlying mechanisms of interaction, but has proven extremely difficult mainly because of the limits in instrumentation. Therefore, most soil carbon models do not consider such a spatially explicit approach or rely on assumptions^21^. Fractionation methods including disaggregating treatments, dispersion, density separation and sedimentation have often been used in various combinations to study the dynamics of SOM fractions^22,23^ but do not necessarily yield spatial information about the location of OM within the soil pore network. Microscopy, e.g. optical microscopy, transmission electron microscopy^24^ or scanning electron microscopy allow visualization of the pore structure but only in two dimensions (2D). Three dimensional (3D) information on submicron pore structure and OM compartments can be obtained via focused ion beam scanning electron microscopy (FIB-SEM), however, this is a destructive technique^25,26^. In contrast, X-ray computed tomography^27^ is a non-destructive technique that allows the investigation of the inner structure of undisturbed soils. De Gryze et al.^28^ were among the first to successfully discriminate added POM in X-ray CT images of undisturbed aggregates. However, manual pinpointing was required because of low image contrast, introducing subjectivity in the data analysis and interpretation. Poor OM contrast in CT volumes is typically attributed to its broad grey value interval that overlaps with both the pore space and the mineral phase intervals. Additionally, partial volume effects complicate detection of finer OM with dimensions close to the voxel resolution. High quality images in combination with a sufficiently fine spatial resolution are thus required for (semi-)automated X-ray CT based discrimination of OM. Both image quality and resolution depend on sample characteristics, X-ray CT scanner settings and scan settings. Considerable variability can be introduced by the choice of threshold methodology and operator influence on the segmentation process^29^. Therefore, the development of automated and standardized OM segmentation protocols, which are less operator biased, could result in more accurate results by taking account of parameters such as POM shape and size, and the spatial patterns of grey values in the POM particles^30^. However, this is limited by poor image contrast of POM from conventional X-ray CT systems.

Recent research has demonstrated that artificially raising the attenuation of OM through binding with added heavy-elements may increase CT-contrast and facilitate POM segmentation in X-ray CT imaging^31,32^. Heavy-element based staining has been used by Chenu and Plante^24^ to discriminate SOM from mineral soil particles in transmission electron microscopy of thin soil sections (2D). Peth et al.^31^ used osmium tetroxide (OsO_4_) as it is known to react with unsaturated C-bounds, which are omnipresent in organic matter (e.g. in lipids, proteins). They visualized POM in aggregates using 3D with synchrotron-based X-ray microtomography via the absorption edge scanning technique, facilitated by its typical monochromatic X-ray beam. Although an appropriate method for the identification of POM in soil, OsO_4_ is extremely toxic, reactive and highly volatile, thus posing serious health and environmental risks^33^. In addition, the availability of synchrotron facilities is limited thereby restricting permanent access. In contrast, availability of so called ‘benchtop’ and laboratory based X-ray µCT scanner systems is rapidly increasing. Van Loo et al.^32^ therefore evaluated a wide variety of chemical staining agents to selectively increase the attenuation of POM particles in mixtures with sand using a lab-based CT scanner. The four most promising POM staining agents were lead nitrate (Pb(NO_3_)_2_), lead acetate (PbAc), phosphomolybdenic acid (PMA) and silver nitrate (AgNO_3_). The risk of unintended staining of the mineral soil fraction in natural soils is potentially higher using these reagents because of the increased number of reactive surfaces in the silt and clay fractions, thereby highlighting the need to identify selective staining agents for the reactive sites of POM to avoid misclassification.

In this study, we further tested the potential of these four chemical staining agents (Pb(NO_3_)_2_, PbAc, PMA, AgNO_3_) to selectively increase CT-contrast of POM particles in mixtures of sand combined with fine silt, coarse silt or clay. We assessed the differentiation in shifts of CT-grey values of POM and mineral fractions following staining using a lab-based X-ray µCT scanner. SEM–EDX was used to ascertain selective staining of POM vs. mineral particles. We also checked potential changes in the physical structure of the ‘soil’ mixtures caused by the addition of these staining agents as aqueous solutions. Finally, the increase in POM contrast was compared to that obtained by the use of gaseous OsO_4_.

## Materials and methods

### Sample collection and preparation

#### Mineral particle size fractions

The test materials, a number of artificial soil mixtures, were prepared using varying fractions of sand, silt and clay, but with a fixed mass percentage of POM. Mineral particle size fractions were derived from a Pleistocene niveo-ealian deposited soil (7% clay, 42% silt, 51% sand; pH_H2O_ = 6.3; 0.797% SOC; 0.061% N), collected from the plough layer (0–30 cm) of a cropland field in Lendelede (Belgium). A bulk soil was dry sieved using 2000, 200 and 53 µm sieves. The resulting soil fractions were dispersed by shaking with a 50 g l^−1^ sodium metaphosphate (1:3 w v^−1^ ratio) solution, sieved again and rinsed with deionized water to produce three size fractions: coarse sand (cSa: 200–2000 μm), fine sand (fSa: 53–200 μm) and silt + clay (S + C: < 53 μm). The cSa and fSa fractions were then heated in a muffle furnace at 500 °C for 5 h to remove sand sized OM.

Gravity sedimentation in water based on Stokes’ law (in 30 cm high water columns) was used to separate the S + C fraction into three particle size fractions. Briefly, we first separated clay (C: < 2 µm), followed by fine silt (fSi: 2–20 µm) by aspirating the suspensions which were free of any coarser textured particles. This was repeated for up to 10 consecutive sedimentation cycles. Coarse silt (cSi: 20–53 µm) was then collected as final sediment. The clay and fine silt suspensions were concentrated by water evaporation in 1 L beakers on a hot plate. In order to minimize the effects of OM removal on mineralogy, the OM associated with these three fractions was removed by oxidation with NaOCl rather than by combustion, as follows. Subsamples were weighed into Nalgene centrifuge tubes and a 156.8 g l^−1^ active chlorine solution (at pH 8) was added to a ratio of 1:10 soil:NaOCl-solution and the suspensions were rotary-shaken for 6 h. After centrifugation, the supernatant was removed and the process was repeated twice. Any remaining NaOCl was removed by the addition of deionized water to the centrifuge tubes, shaking for 1 h, centrifugation and decanting. After repeating this ‘rinsing’ step five times, the mineral fractions were dried at 30 °C.

#### Particulate organic matter

Discrete OM particles, originating from non-woody plant structures (e.g. cover crops, crop residues, manure), were isolated from a sandy loam soil (4.8% clay, 26.2% silt, 69.0% sand; 2.53% OC) collected from the plough layer of a cropland field in Liezele (Belgium). After dispersion of the soil via soft ultra-sonication (60 J ml^−1^) in deionized water (ratio 1:4), mineral particles > 53 µm and POM > 53 µm were separated from the bulk soil via wet sieving on a 53 µm sieve. In order to separate POM particles from the remaining < 53 µm mineral fraction, POM was extracted using gravity sedimentation in water columns (30 cm high) according to Stokes’ law. After drying the POM at 50 °C, the POM was sieved using a 2 mm sieve to retain only the 53–2000 µm OM particles. Scanning electron microscopy (SEM) was used to ascertain the purity of the POM and revealed limited remnant mineral particles attached onto the surface of the POM (vide infra).

#### Preparation of soil samples

**Figure 1:**
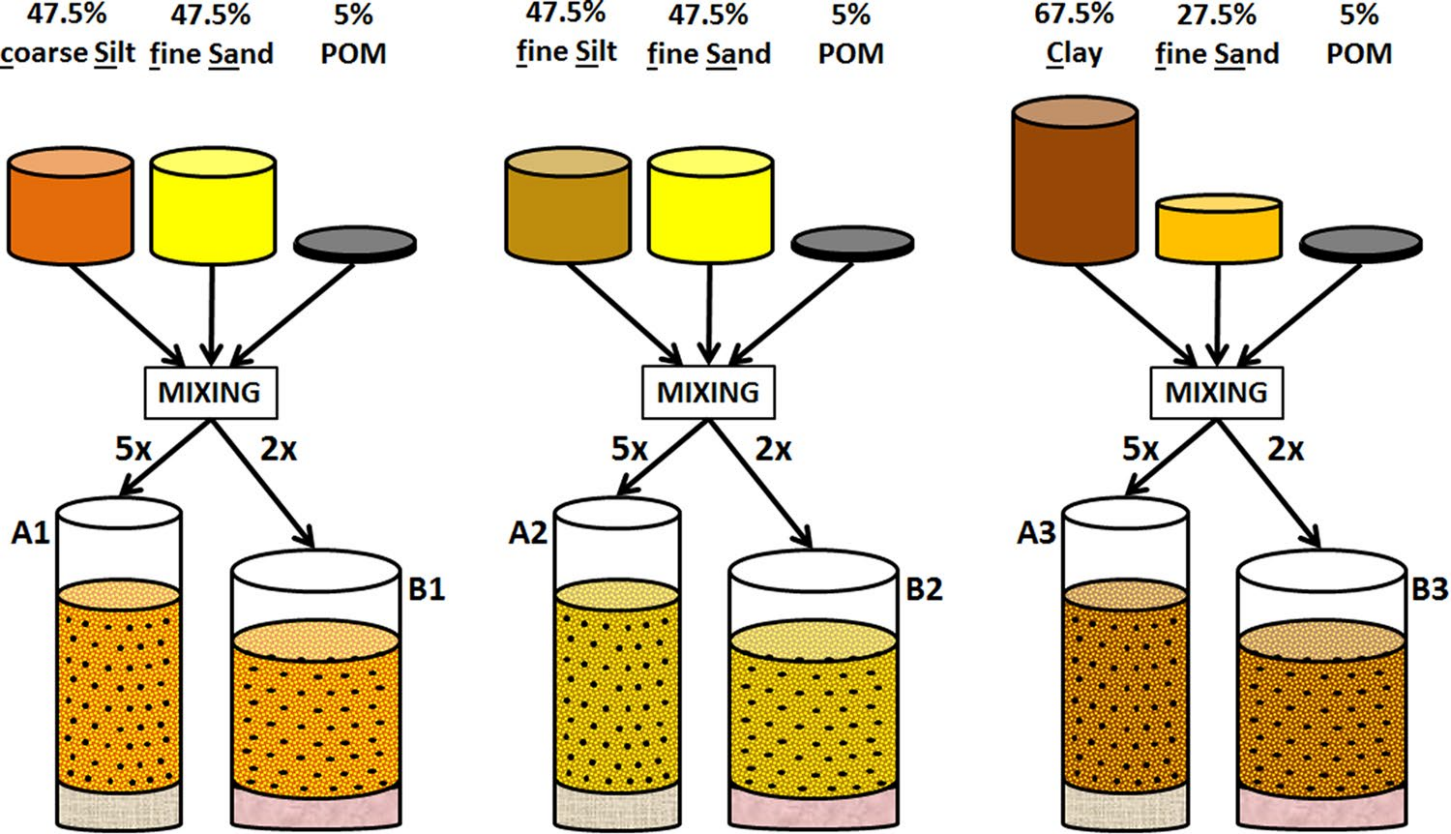
Schematic overview of soil sample preparation for staining experiments. Three series of five soil samples were constructed in micro-barrier sealed plastic tips (A1-3) containing a 0.45 µm nylon filter (grey layer). Each series consisted of 5% (mass) POM combined with a different mineral mixture. Four samples of each series were stained with a different staining agent (i.e. silver nitrate [AgNO_3_], lead nitrate [Pb(NO_3_)_2_], lead acetate [PbAc] and phosphomolybdenic acid [PMA]), and one with deionized water (unstained control). Three identical series of two soil samples (B1-3) were constructed in centrifuge tube filter inserts, provided with a 0.22 µm pore diameter filter (pink layer). One sample of each series was stained with OsO_4_, the second sample of each series functioned as unstained control.

Small soil columns (0.50 cm in diameter × 0.65 cm high) were constructed in micro-barrier sealed plastic centrifuge tips (MultiGuard barrier tips,  < 5 µm pore diameter) containing a 0.45 µm nylon filter (Fig. 1). Three series of 5 soil mixtures were prepared, each with 5% POM (mass%) in combination with different mineral mixtures: (1) fSa (47.5 mass %) and cSi (47.5 mass %); (2) fSa (47.5 mass %) and fSi (47.5 mass %), or (3) fSa (67.5 mass %) and C (27.5 mass %).

A different recipient (Costar Spin-X Centrifuge Tube filter inserts, 0.22 µm pore diameter) was used to prepare artificial soil mixtures (0.60 cm in diameter × 0.45 cm high) to be fumigated with OsO_4_ vapour (Fig. 1).

### Staining procedure

#### Pressurized perfusion of staining solutions

The soil samples of each series in the MultiGuard barrier tips were perfused with four different staining solutions and with deionized water (unstained control). The staining agents were silver nitrate (AgNO_3_), lead nitrate (Pb(NO_3_)_2_), lead acetate (PbAc) and phosphomolybdenic acid (PMA) as these were previously selected by Van Loo et al.^32^ as most appropriate for staining natural OM. The staining solutions (all at 1 M) were perfused through the soil samples according to the procedure described by Van Loo et al.^32^. In summary, pressurized perfusion was achieved by establishing an under pressure at the lower side of the sample holders by connecting a membrane vacuum pump. First, the soil samples were perfused with 20 ml of deionized water, followed by 5 ml of the staining solution. The vacuum pump was then stopped and 5 drops of water were added to prevent crystallization during a subsequent 24 h reaction period. The soil samples were then rinsed by perfusion with 50 ml of deionized water, and dried at 20 °C in a fume hood for 48 h.

#### Osmium staining via diffusion

One soil column from each artificial soil was stained with OsO_4_, following Rawlins et al.^33^. Because of the toxicity, reactivity and volatility, strict health and safety protocols were adhered to but not described in full here. Inside a fume hood, 0.5 ml OsO_4_ was pipetted into the bottom of a Costar Spin-X Centrifuge Tube vial. The filter inserts were placed into the vials and sealed with caps. The OsO_4_ was left to diffuse through the soil sample for 48 h and the filter inserts were then removed. On removal from the centrifuge tubes the inserts were shortly ventilated and wiped clean using maize oil in order to neutralize health risks. Finally, the inserts were placed in new vials and the caps sealed. A second soil column of each of the three soil types was constructed in a separate filter insert but not stained and served as control treatment.

### X-ray µCT scanning and image reconstruction

X-ray µCT scanning was performed with an in-house developed X-ray computed tomography scanner^34^ and controlled by in-house developed software^35^ at the Centre for X-ray Tomography of Ghent University (UGCT, http://www.ugct.ugent.be). The Feinfocus FXE-160.50 directional X-ray tube was operated at 100 kV and 140 µA. In total 1801 projections with an exposure time of 1000 ms were recorded via a Varian Paxscan 2520 detector.

In-house developed Octopus Reconstruction software^36^ (distributed by XRE, Ghent, Belgium, http://www.xre.be) was used to reconstruct the raw data into a 16-bit greyscale dataset. This resulted in 21 CT volumes of 1450 × 1450 × 1633 cubic voxels with 7 µm voxel pitch.

### Evaluation of staining agents

X-ray attenuation grey value distributions of the different bulk soil mixtures were firstly represented by histograms. For the different peaks in the composite frequency distribution an in-house developed algorithm was used to fit Gaussian distributions for each of the soil (mineral) phases present. The magnitude of an eventual peak shift to higher grey values is then indicative of enhanced X-ray attenuation caused by binding of the staining agent onto the specific soil phase.

The staining-caused shift in X-ray attenuation of POM was evaluated by comparing grey value distributions of 20 to 25 selected POM particles per soil column. Particles were visually identified with the AVIZO (http://www.fei.com) software package based on their grey value pattern and shape. A region of interest was then ‘pin-pointed’ in these selected POM particles, excluding the edges to rule out voxels suffering from the partial volume effect.

Scanning electron microscopy with energy-dispersive X-ray spectroscopy (SEM–EDX) was used to additionally verify the selectiveness of staining agents for POM versus mineral particles. Seven subsamples of selected staining agent—POM mineral phase mixture combinations were scanned with a TESCAN Integrated Mineral Analyzer (TIMA) high resolution SEM. Minerals and POM could be readily visually recognized but EDX-based detection of C and Si was always used to ascertain this as well. To enable EDX detection of the staining elements (Mo, Pb and Ag) the samples were intentionally not coated with gold or carbon.

### Pore volume

Soil porosity was determined and compared between stained and unstained samples as a first evaluation of possible staining-caused alterations to soil structure. The CT-visible pore volume (pore diameter > 14 µm) was determined after segmenting pore space with the Octopus Analysis software (XRE, http://www.xre.be). A conservative histogram-based single grey value threshold was applied.

## Results and discussion

### Staining of POM in presence of fine sand and coarse silt

**Figure 2:**
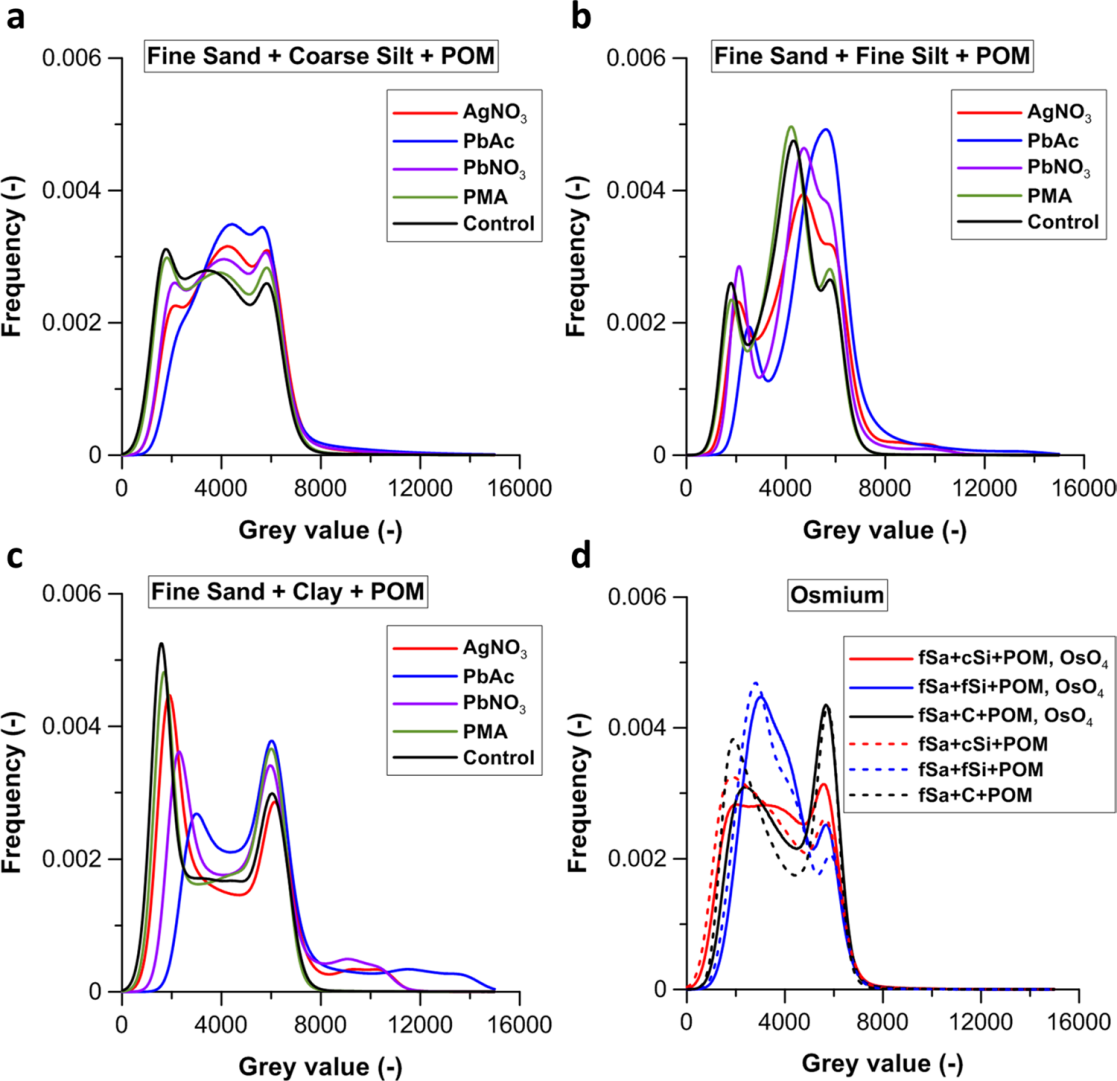
Grey scale histogram (0–16,000) for 16 bit images of (**a**) the fine sand + coarse silt + POM soil samples, (**b**) the fine sand + fine silt + POM mixtures and (**c**) the fine sand + clay + POM mixtures. The unstained control treatment (black) and the stained AgNO_3_ (red), PbAc (blue), Pb(NO_3_)_2_ (purple) and PMA (green) treatments. (**d**) Grey scale histogram (0–16,000) for 16 bit images of OsO_4_ stained fine sand + coarse silt + POM (fSa + cSi + POM; red), fine sand + fine silt + POM (fSa + fSi + POM; blue) and fine sand + clay + POM (fSa + C + POM; black) mixtures. The equivalent control treatments are indicated by dotted lines.

**Figure 3:**
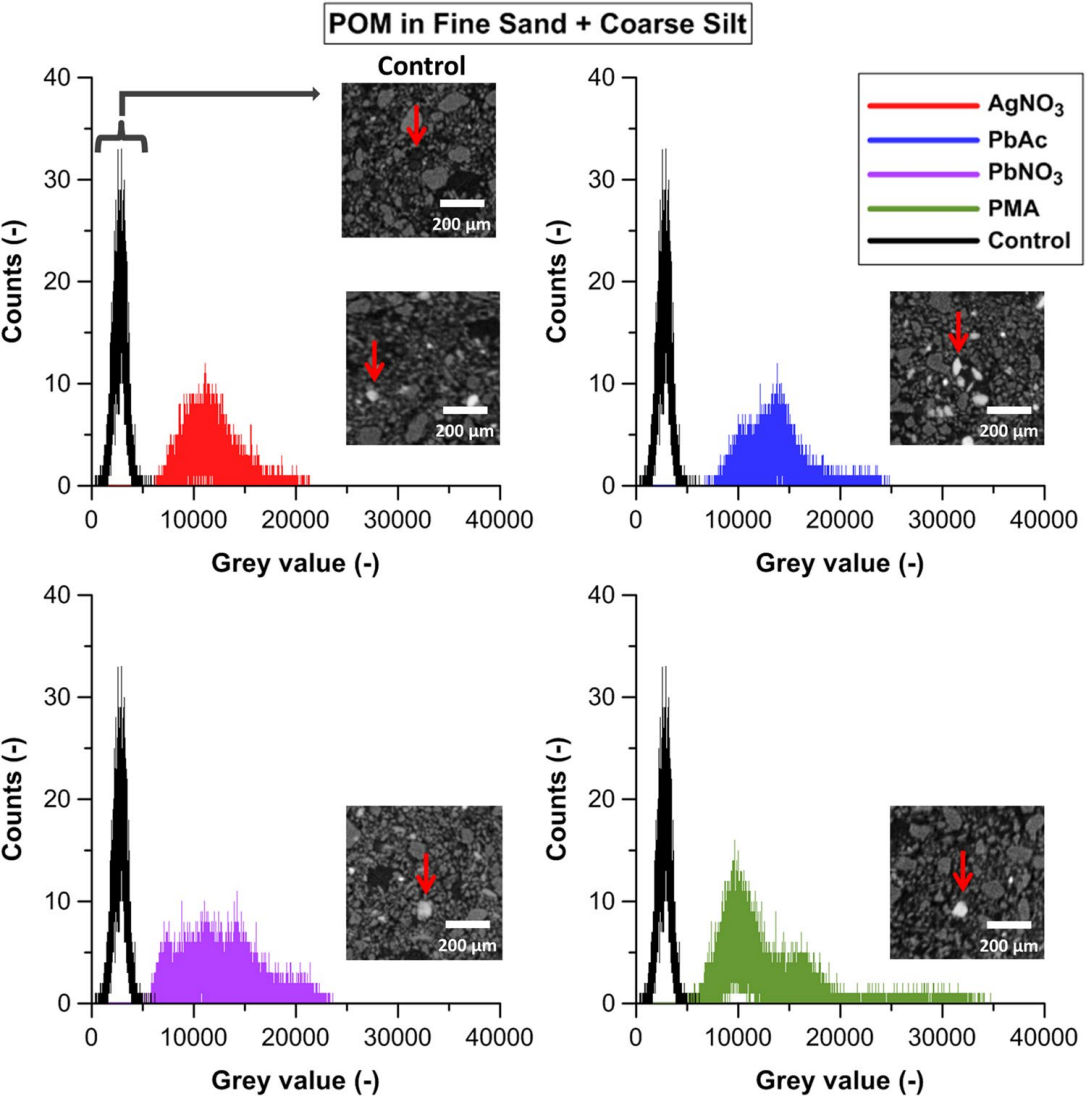
Grey scale histograms (0–40,000) of operator-supervised pin-pointed POM particles in 16 bit images of fine sand + coarse silt + POM mixtures: the unstained control treatment (black) and the stained AgNO_3_ (red), PbAc (blue), Pb(NO_3_)_2_ (purple) and PMA (green) treatments. For each treatment, a two dimensional grey scale image representing a segment of a horizontal slice of the fine sand + coarse silt + POM mixtures is included (image contrast was enhanced in this figure). An unstained POM particle in the control treatment and stained POM particles in the AgNO_3_, PbAc, Pb(NO_3_)_2_ and PMA treatments are indicated by the red arrows.

Compared to the unstained control (unstained sample mean peak grey value ± standard deviation: 3686 ± 1557), all staining treatments caused a slight shift of the central grey value peak to the right, towards 3757 ± 1272 and 4421 ± 1646 for the PMA and PbAc treatments, respectively. This central peak represents X-ray attenuation by coarse silt and POM (Fig. 2a). However, no increased X-ray attenuation was observed in coarse silt in the reconstructed CT-sections (Fig. 3). Instead, the grey value distributions of pin-pointed POM particles (Fig. 3) confirmed that staining did cause a clear shift in POM’s grey values, viz. from 1000–4000 to 6000–18,000. This contrast enhancement of POM was more intense for AgNO_3_, PbAc and Pb(NO_3_)_2_ treatments compared to PMA (Fig. 3) and caused the very clear increase in the histogram’s right tail (grey values > 7500), which was absent with PMA treatment (Fig. 2a). Staining with Pb^2+^ or Ag^+^ also resulted in broad grey value peaks of POM that overlapped those of the coarse silt (grey value range: 2500–5000) and fine sand particles (grey value range: 5000–7500), represented by the second and third peak on the bulk soil histograms (Fig. 2a). However, the histogram peak maxima of the AgNO_3_ (11,118), PbAc (13,829), Pb(NO_3_)_2_ (14,255) and PMA (9678) stained POM particles (Fig. 3) clearly exceeded the grey value range of both the coarse silt and fine sand, which suggests that CT grey value based discrimination of POM from these mineral fractions should be feasible. The smaller peak shift in the POM histograms for PMA (Fig. 3) shows that PMA was less efficient in increasing attenuation of POM and this could be ascribed to the lower atomic mass (AM) of Mo (95.9) compared to Pb (207.2). Likewise, the effect of Ag (AM 107.9) was closer to that of Mo. Next to atomic mass, the affinity for binding to OM likely also differs between the contrast agents. While Ag^+^ and Pb^2+^ bind with the organic functional groups of POM via ionic bonds, PMA is known to interact with conjugated unsaturated bonds^37^.

There was no shift towards higher grey values in the third peak in the soil core histograms, representing fine sand (Fig. 2a). In addition, we did not find traces of Pb, Ag or Mo on any SEM–EDX scanned fine sand or coarse silt particles (Supplementary Fig. S1). Visual inspection of horizontal reconstructed slices revealed a clear discrimination of the stained POM particles vs. the soil mineral phase. SEM–EDX scans confirmed substantial Mo, Pb or Ag EDX-peaks on randomly chosen POM particles, directly confirming the successful selective staining of POM by all agents (Supplementary Fig. S1). Smaller mineral patches on the surface of POM were visible in the SEM-images (Supplementary Fig. S2), but our analysis demonstrates that these were not responsible for Mo, Pb or Ag-staining of the POM. Stained POM could also be discriminated from several other high density particles such as glauconite, since the latter were not only characterized by higher grey values^32^ but also a different grey value pattern. The SEM–EDX spectra of some dense mineral particles moreover revealed Zr peaks, suggesting these to be ZrO_2_ or Zr-silicate. In conclusion, the experiment demonstrates that Pb(NO_3_)_2_, AgNO_3_ and PbAc are able to selectively bind with POM in fine sand + coarse silt mixtures. However the potential to raise POM contrast in such soil mixtures by treatment with PMA is smaller.

### Staining of POM in presence of fine silt and clay

**Figure 4:**
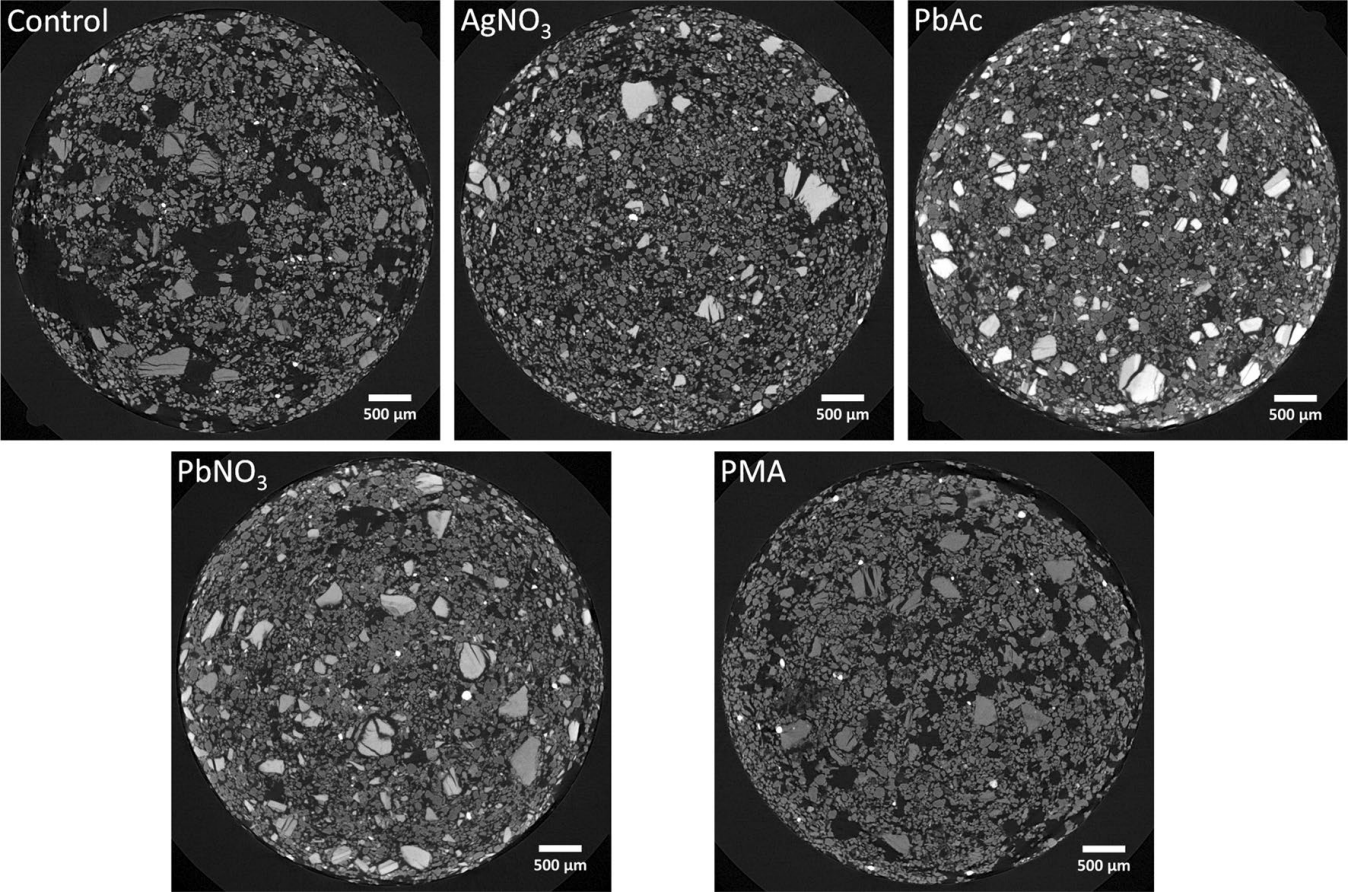
Two dimensional grey scale image representing a horizontal slice of the fine sand + clay + POM mixtures: the control treatment and the stained AgNO_3_, PbAc, Pb(NO_3_)_2_ and PMA treatments (image contrast was enhanced in this figure). Clusters of stained clay particles are clearly observable as brighter structures in the AgNO_3_, PbAc and Pb(NO_3_)_2_ treatment.

PMA did not appear to stain fine silt, with no shifts of peaks in the bulk soil histogram (Fig. 2B), no visible impact on contrast of the soil mineral phase (Supplementary Fig. S3) and again no discernible Mo peaks in the SEM–EDX spectra of mineral particles (Supplementary Fig. S4 and Supplementary Fig. S5). In contrast, AgNO_3_, PbAc and Pb(NO_3_)_2_ appeared rather non-selective for POM (Fig. 2B). Indeed, the staining increased the grey values of histogram peaks corresponding to fine silt (Fig. 2b), which can be seen from shifting fine silt histogram peaks from a grey value of 4303 to 4702–5577. The increase of the fine silt grey values following staining even resulted in a partial (Pb(NO_3_)_2_ and AgNO_3_) or complete (PbAc) overlap with the sand fraction histogram peak. The grey value histogram right tail was also much enlarged by Pb(NO_3_)_2_, AgNO_3_ and PbAc treatment and increasingly overlapped with the grey value range of stained POM (6000–18,000) (Fig. 3). This overlap was clearly an effect of staining of fine silt, as was also apparent in the CT-sections (Supplementary Fig. S3). The shift in histogram grey values following staining in the fine sand + clay mixtures was similar to that in the sand + fine silt mixtures, but with a larger shift in the right tail of the Pb^2+^ and Ag^+^-histograms to grey values between 7500 and > 12,000 (Fig. 2c). However, we did not detect Pb or Ag EDX peaks on pinpointed fine silt or clay particles (Supplementary Fig. S4). But as inspection of CT-sections (Fig. 4) demonstrated that large patches of the mixtures were stained by AgNO_3_, PbAc and Pb(NO_3_)_2_, it is well possible that such discrete stained areas were coincidently not sampled in our ancillary unsystematic SEM-analysis of subsamples. Regardless, overlap in grey value-ranges of Pb^2+^ and Ag^+^ stained POM and stained clay particles clearly impedes proper segmentation of both phases. As was the case for fine silt, PMA treatment did not cause any shift in histogram peaks of mineral particles (Fig. 2c) and there was no visible impact on mineral phase attenuation in the CT-sections (Fig. 4). This also suggests that Mo has a higher potential than Pb^2+^ and Ag^+^ to bind to OM solely and not to mineral surfaces. Indeed, this may be a result of the neutral Mo(VI)O_3_ in PMA, while the cations Pb^2+^ and Ag^+^ may adsorb to negatively charged mineral surfaces. However, Chenu and Plante^24^ did not detect any sorption of both Pb and Ag on pure clay minerals (vermiculite, illite, kaolinite). Despite minerals identified in our clay fraction being kaolinite and smectite, sorption of Pb (as observed by Chenu and Plante^24^) or Ag onto Al and Fe oxides and hydroxides in the clay fraction could have occurred.

While manual pin-pointing of the PMA stained POM was not impeded, automated POM extraction from the CT volume may still prove to be challenging due to the lack of image contrast between POM and mineral soil particles. Very recent work by Lammel et al.^38^ applied a machine learning segmentation tool in synchrotron-based soil CT volumes but experienced limited success. Piccoli et al.^39^ suggested that an operator-based ability for the selection of thresholds may still result in the most accurate segmentation of POM in soil.

### Impact on sample structure

**Figure 5:**
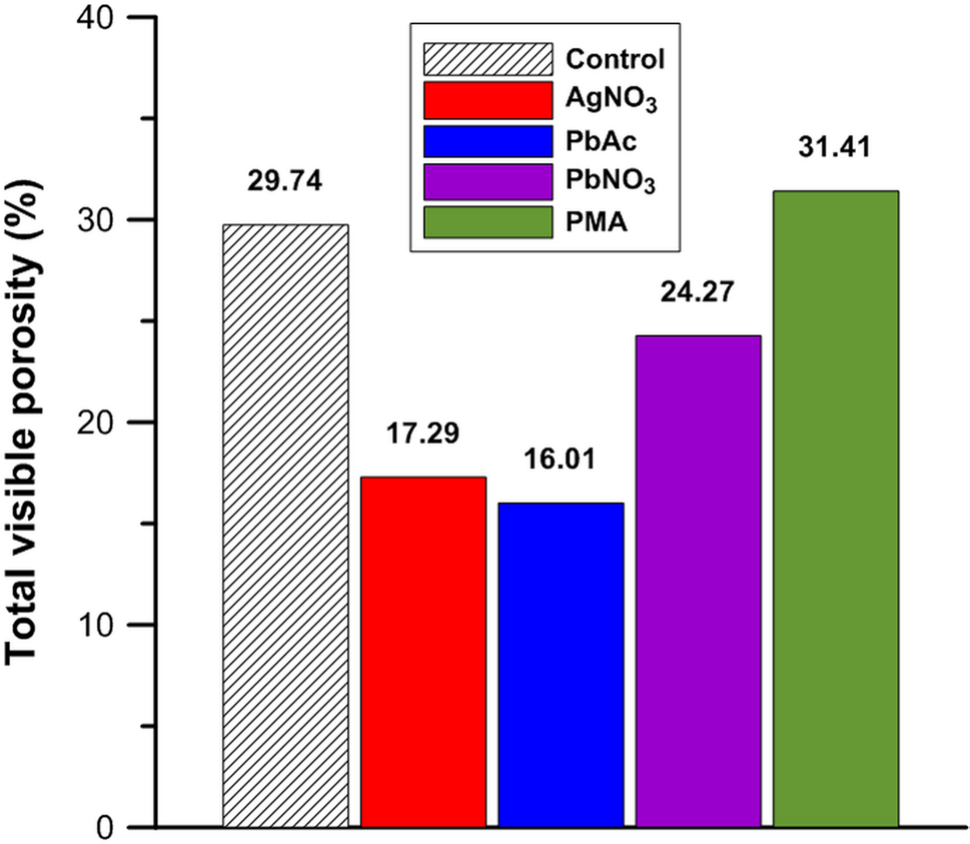
Total X-ray µCT visible porosity in the fine sand + coarse silt + POM mixtures: the unstained control treatment (black) and the stained AgNO_3_ (red), PbAc (blue), Pb(NO_3_)_2_ (purple) and PMA (green) treatments.

Bulk sample histogram evaluation (Fig. 2a) of the fine sand + coarse silt samples demonstrated that the grey value peak of the pore space (unstained sample mean peak grey value ± standard deviation: 1765 ± 609) had slightly shifted following staining with Pb(NO_3_)_2_ (1930 ± 386), AgNO_3_ (1923 ± 392) and PbAc (2204 ± 314), and to a lesser extent with PMA (1801 ± 611). There was also a reduction in the pore space peak height for the Pb(NO_3_)_2_, AgNO_3_ and PbAc stained treatments of 16%, 27% and 42%, respectively. In the PbAc treatment, the pore space peak disappeared nearly completely, whereas this drop was smaller for AgNO_3_ and much smaller for Pb(NO_3_)_2_. In contrast, no such decline in pore space peak was observed in the PMA-stained samples. A decrease in pore volume (Fig. 5) for the Pb(NO_3_)_2_, AgNO_3_ and PbAc treatments matched the trend in reduction of pore peak height. In addition, opposite trends of peak height increases existed for the second, coarse silt peak (+ 6%, 13%, 25%, respectively) and for the third, fine sand peak (+ 18%, 19% and 33%, respectively). Combined, these observations demonstrate that the observed changes in pore space and mineral phase peak areas are the result of the compaction of the samples by the Pb(NO_3_)_2_, AgNO_3_ and PbAc treatments.

Given that peak height reductions were very different for Pb(NO_3_)_2_ and PbAc, the deterioration of soil structure could not be solely related to the heavy-element applied (both containing Pb^2+^). In addition, the decreased porosity is unlikely to have been predominantly due to the addition of NO_3_^−^, as the addition of Pb(NO_3_)_2_ would have impacted structure to a greater extent than AgNO_3_ which contains less (0.5×) NO_3_^−^ (solutions were all at 1 M). Deterioration of the sample structure was therefore more likely to be a physical phenomenon. One possible mechanism could be that an increased underpressure, as a result of perfusion of a more viscous agent, could have more severely disrupted the structure of the soil mixtures. However, an increasing degree of structure distortion could not be related to a higher viscosity of the staining solutions (considered at 0.01 or 0.1 M^40–42^). Hence, the results in this study did not allow identification of the exact origin of this artifact. As a consequence, caution has to be taken when perfusing soil samples with liquid staining agents since deterioration of soil structure would alter spatial location of the POM as well. Thus, these results indicate that soil structure validation is still required as long as the specific cause for deterioration is not identified.

The Pb(NO_3_)_2_, AgNO_3_ and PbAc treatment histograms of both the fine silt (Fig. 2b) and clay (Fig. 2c) mixtures also had a smaller and broader pore space peak. It is likely that these are both derived from increased occurrence of partial volume effects (PVE) from chemical staining. It is thought that compaction following staining resulted in more voxels containing both pore space and mineral particles. With a voxel resolution of 7 µm this increased the number of pore space voxels with an intermediate grey value (2000–4000). The order in grey value increase magnitude for the fine silt and clay samples was similar as for the coarse silt mixtures: PbAc > Pb(NO_3_)_2_ > AgNO_3._ Chemical staining had an increasingly stronger effect on pore space X-ray attenuation for the finer particle size mixtures, probably because of more intensive binding of Ag^+^ and Pb^2+^ on their much larger reactive surfaces.

In this study the combination of the structural degradation and the overlap in grey value ranges of POM and the mineral fractions render these staining agents unsuitable for staining natural soils. However, further development and testing of the staining method may reduce the impact on soil structure to a minimum. PMA treated silt and clay samples were not structurally degraded and no undesirable shifts of mineral particles’ grey value ranges were observed. However, the artificial soil samples are not fully representative of naturally structured soil and further testing could also rule out degradation of natural soil structure by perfusion with PMA solutions. We expect structural integrity of natural soil samples to exceed that of the ‘loose’ soil mixtures tested in this work.

### Performance of PMA compared to gaseous OsO_4_ staining

Inspection of horizontal CT-sections suggested that OsO_4_ (Supplementary Fig. S6a–c) did not increase X-ray attenuation of mineral material. This is also suggested by the absence of a shift to the left of mineral fraction peaks (grey value 2500–7000), following OsO_4_ treatment (Fig. 2d).

**Figure 6:**
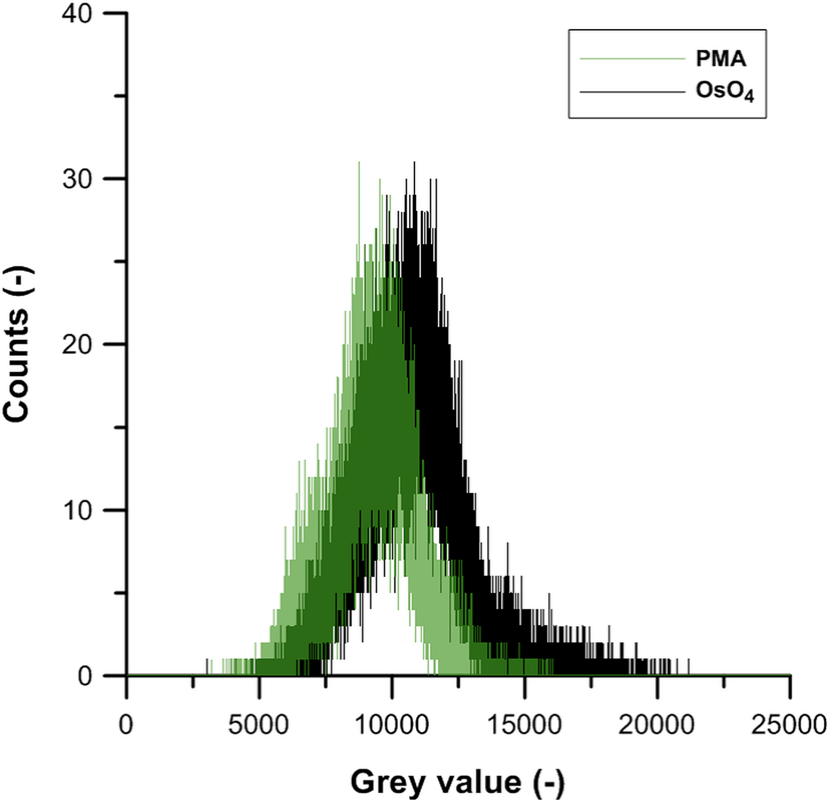
Grey scale histogram (0–25,000) for pin-pointed POM particles in the 16 bit images of PMA (green) or OsO_4_ (black) stained fine sand + clay + POM mixtures.

The grey value distribution obtained via manual pin-pointing of POM particles demonstrated that the grey value interval of OsO_4_-stained POM (Fig. 6) corresponded to the right tail of the soil mixture histograms. This effect was larger but still comparable to the POM grey value shift obtained by PMA treatment. This outcome is a likely result of both staining agents targeting unsaturated bonds in e.g. hydrocarbons or proteins, and the higher atomic mass of Os (190.2) compared to Mo (95.9). Because of the significant health risks when using OsO_4_, PMA appears to be a suitable alternative. The increase in POM grey values following both PMA and OsO_4_ staining showed a sufficient differentiation of POM particles from the mineral fraction, at least for manual pin-pointing. However, single threshold-based segmentation of PMA- or OsO_4_-stained POM in the X-ray CT volumes obtained with this lab-scale polychromatic X-ray µCT system did not appear possible. More sophisticated segmentation algorithms are required, as was also very recently suggested by Piccoli et al.^39^. We propose to develop self-learning algorithms (e.g. incorporate machine learning) that consider the local grey value patterns of POM in combination with morphological characteristics for a more objective and faster segmentation of the stained POM. By using self-learning algorithms, the expert intervention to segment POM particles would be reduced to an absolute minimum and decrease further over time. In addition, technological development will very likely further enhance the X-ray CT resolution, which will strongly improve the morphological characterization of finer grained POM.

Alternatively, the occurrence of a K-edge in the X-ray absorption spectrum of molybdenum could be exploited to discriminate POM in CT images. Rawlins et al.^33^ and Peth et al.^31^ have previously used the occurrence of a K-edge in the X-ray absorption spectrum of Os by scanning soil with synchrotron µCT at photon energies immediately below and beyond the K-edge successfully. However, the K-edge of Mo is situated at 20 keV, an energy level at which most X-rays may be attenuated by the soil mineral fraction, thereby probably making a dual energy approach similar to that used for Os challenging for non-synchrotron scanners and probably also for synchrotrons. Very recently, Lammel et al.^38^ identified gaseous iodide (I_2_) as a plausible candidate for selective staining of OM in soil for use with synchrotron scanners. However, they did not fully demonstrate its selectivity for OM versus silt and clay sized mineral particles, nor in X-ray µCT soil volumes obtained with non-synchrotron scanners.

The findings presented here demonstrate that laboratory based X-ray µCT scanners may also enable the segmentation of OsO_4_-stained POM from mineral particles, provided that better segmentation tools are developed. This opens up new possibilities for a more widespread application of the OsO_4_ staining technique due to much better availability of laboratory based X-ray µCT scanners and throughput time of samples.

## Conclusion

This study clearly demonstrates that the potential of the Pb(NO_3_)_2_, AgNO_3_ and PbAc staining agents to increase the X-ray attenuation of POM in CT volumes of soil seems to be limited to sandy soils characterized by a high infiltration capacity. In contrast to cationic staining solutions, staining agents that do not interact via ionic binding but via e.g. interaction with unsaturated double bonds in OM, as is the case for PMA, have promise. Although the associated increases in the grey values of POM allowed for supervised segmentation, the contrast with other soil phases is still insufficient to support currently available methods for (semi-)automated OM segmentation in soil CT volumes. Development of improved segmentation algorithms considering both the grey value pattern and shape of POM can furthermore contribute to more time-efficient and operator-independent POM segmentation.

The compatibility of PMA with natural undisturbed soil should be further explored, and could include further optimization of perfusion techniques and the effectiveness of less concentrated PMA staining solutions. Compared to OsO_4_ staining, perfusion of soil with PMA results in smaller increases in X-ray attenuation of POM when subjected to a polychromatic X-ray beam. Therefore, the investigation of other, non-cationic staining agents can potentially reveal chemicals with a stronger staining efficiency, which may potentially outperform staining with OsO_4_. We conclude that the use of PMA or OsO_4_ staining in non-synchrotron CT-scanning does hold potential to identify discrete OM in soils.

## Supplementary Information

Supplementary Information.

## Data Availability

The datasets generated during and/or analyzed during the current study are available from the corresponding author on reasonable request.
