# Supplementary material for: Chemical staining of particulate organic matter for improved contrast in soil X-ray µCT images
Source: Sci Rep. 2021 Jan 11;11:370. doi: 10.1038/s41598-020-79681-5 (PMC7801699; doi:10.1038/s41598-020-79681-5)
Supplement: Supplementary file 1 — Supplementary Information. [file 41598_2020_79681_MOESM1_ESM.pdf]

Supplementary Material

Scientific Reports

Chemical staining of particulate organic matter for improved contrast in soil X-ray µCT images

Peter Maenhout*, Stefaan De Neve, Joanna Wragg, Barry Rawlins, Jan De Pue, Luc Van Hoorebeke, Veerle Cnudde, Steven Sleutel

*Corresponding author:

Research Group of Soil Fertility and Nutrient Management, Department of Environment, Ghent University, Coupure Links 653, 9000 Gent, Belgium

E-mail address: peter.maenhout@ugent.be

Tel.: +32 9 264 60 57

Supplementary Material:
Chemical staining of particulate organic matter for improved contrast in soil X-ray µCT images
Peter Maenhout, S. De Neve, J. Wragg, B. Rawlins, J. De Pue, L. Van Hoorebeke, V. Cnudde, S. Sleutel

Supplementary Figures:

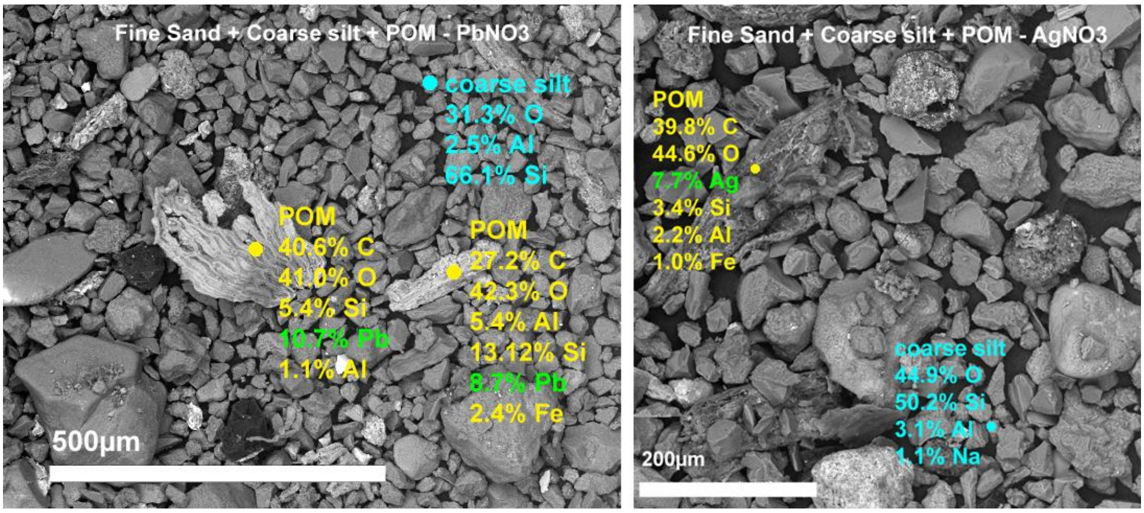

Figure S1: SEM images of fine sand + coarse silt + POM mixtures stained with $PbNO_3$ and $AgNO_3$. The indicated percentages give the weight percentage of main identified elements in EDX-spectra of the neighbouring spot. No traces of Pb or Ag were found on the coarse silt particles, while they clearly emerged in POM EDX-spectra.

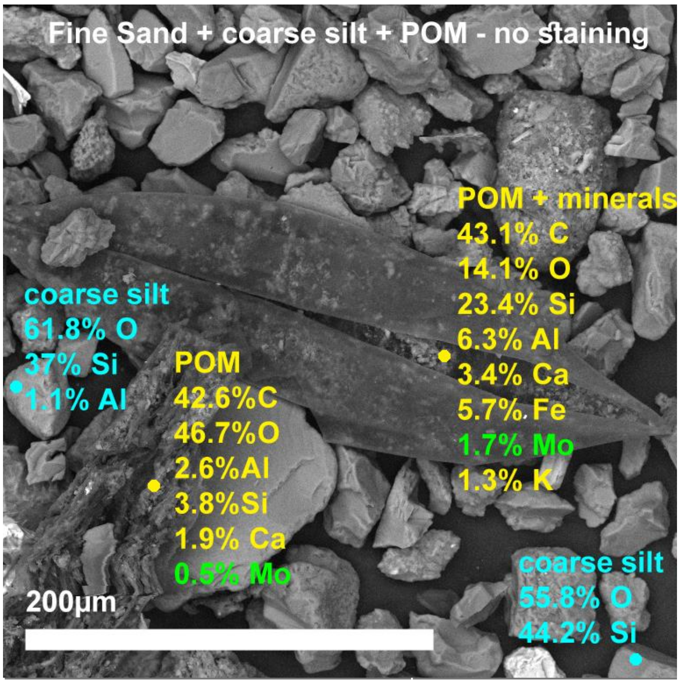

Figure S2: SEM image of one of the fine sand + coarse silt + POM mixtures with no CT-contrast enhancement staining applied. The indicated percentages give the weight percentages of main identified elements in EDX-spectra of the neighbouring spot. Mineral patches on the POM surface of the middle OM particle are confirmed by elevated Si and Al weight percentages. Much less mineral matter was present on the lower left OM particle. Note also the background signal of Mo on these two POM particles.

Supplementary Material:
Chemical staining of particulate organic matter for improved contrast in soil X-ray µCT images
Peter Maenhout, S. De Neve, J. Wragg, B. Rawlins, J. De Pue, L. Van Hoorebeke, V. Cnudde, S. Sleutel

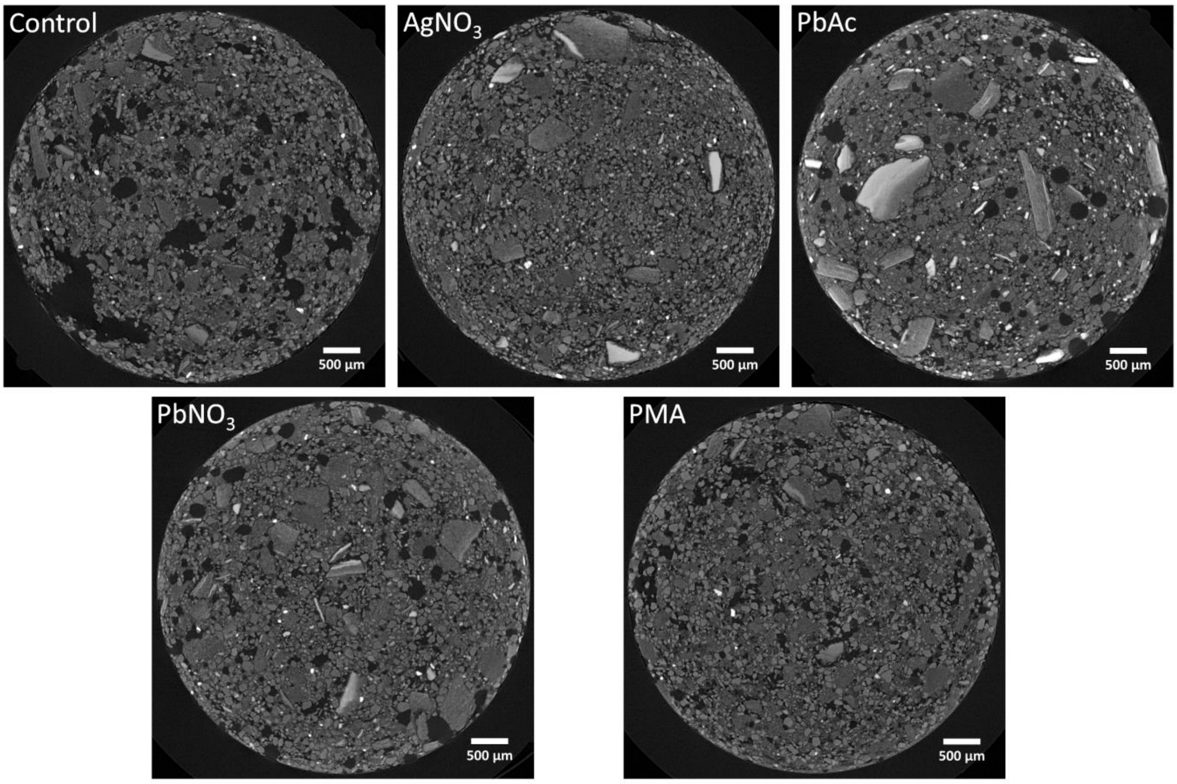

Figure S3: Two dimensional grey scale image representing a horizontal slice of the fine sand + fine silt + POM mixtures: the control treatment and the stained $AgNO_3$, PbAc, $Pb(NO_3)_2$ and PMA treatments (image contrast was enhanced in this figure).

Supplementary Material:

Chemical staining of particulate organic matter for improved contrast in soil X-ray µCT images

Peter Maenhout, S. De Neve, J. Wragg, B. Rawlins, J. De Pue, L. Van Hoorebeke, V. Cnudde, S. Sleutel

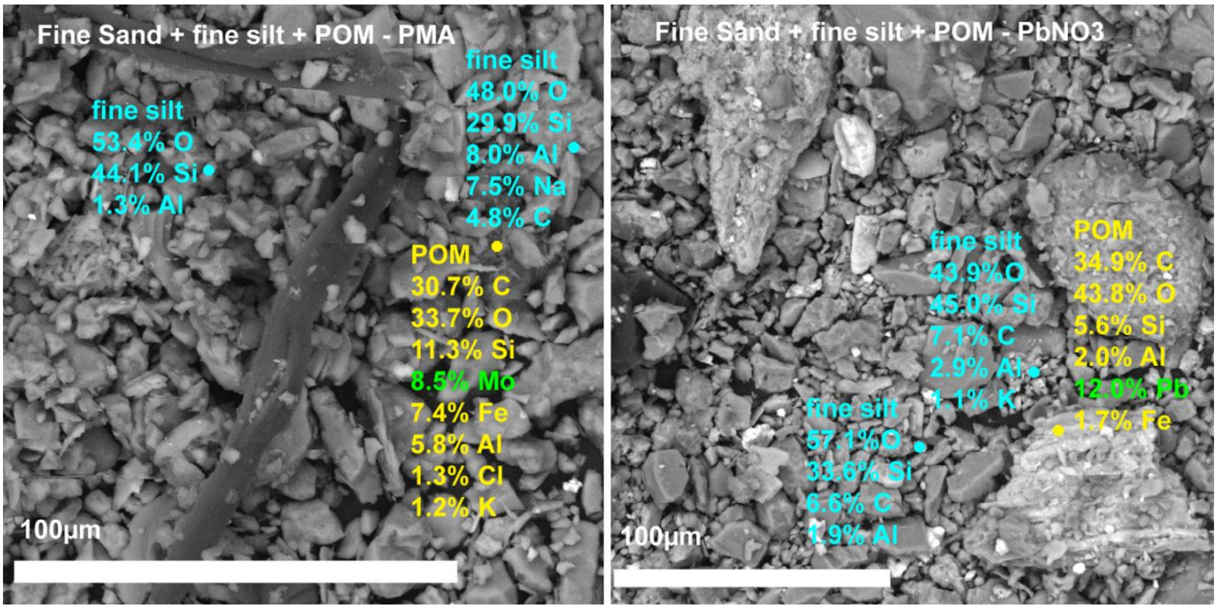

Figure S4: SEM images of fine sand + fine silt + POM mixtures stained with PMA and $PbNO_3$. The indicated percentages give the weight percentage of identified elements in EDX-spectra of the neighbouring spot. No traces of Mo or Pb were found on the fine silt particles, while they clearly emerged in the POM EDX-spectra. Note that some of the (pre-ashed) mineral particles contained C, an artefact caused by placement of the sample onto an organic carrier into the SEM.

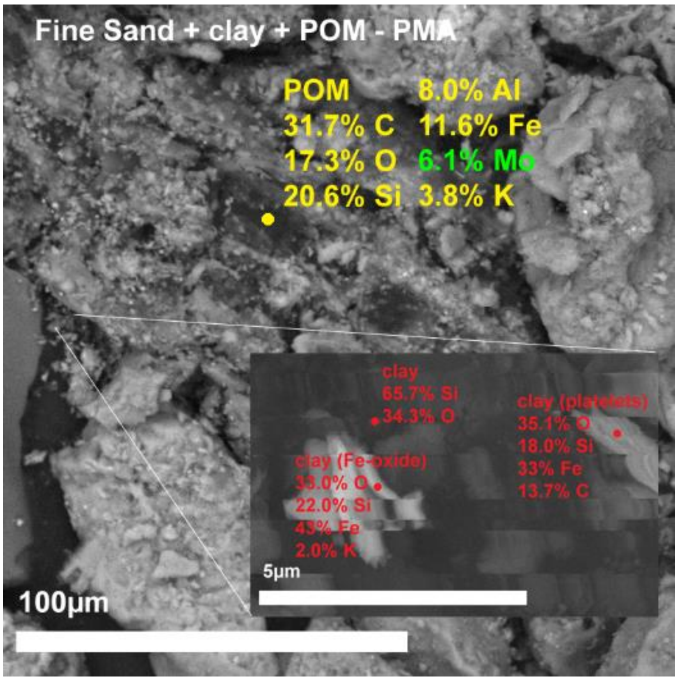

Figure S5: SEM image of one of the fine sand + clay + POM mixtures stained with PMA and a clos-up of some clay particles. The indicated percentages give the weight percentage of identified elements in EDX-spectra of the neighbouring spot. No traces of Mo were found on the three clay particles (presumably an Fe-oxide, a quartz particle and a bundle of clay platelets), while Mo clearly emerged in EDX-spectrum of POM.

Supplementary Material:

Chemical staining of particulate organic matter for improved contrast in soil X-ray µCT images

Peter Maenhout, S. De Neve, J. Wragg, B. Rawlins, J. De Pue, L. Van Hoorebeke, V. Cnudde, S. Sleutel

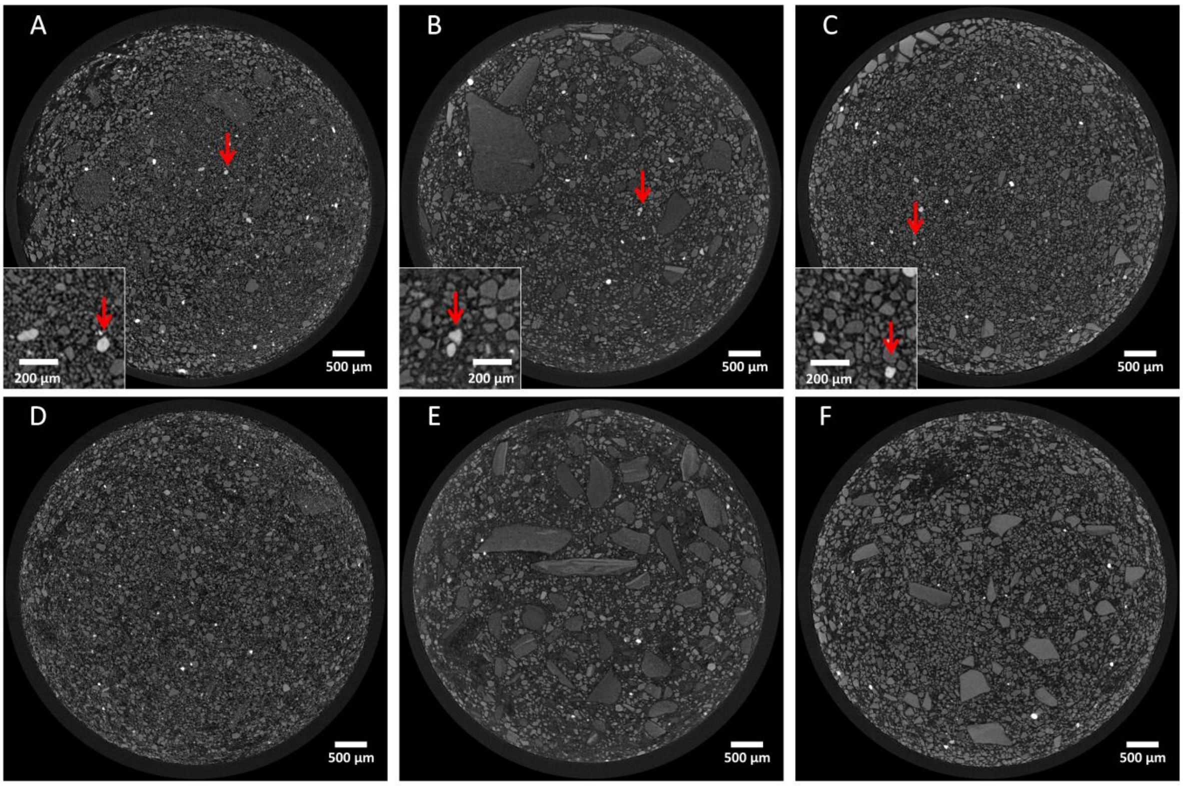

Figure S6: Two dimensional grey scale image representing a horizontal slice of OsO_4 treated fine sand + coarse silt + POM (A), fine sand + fine silt + POM (B) and fine sand + clay + POM (C) mixtures. Stained POM particles are indicated by red arrows and are magnified within the white frames. Images of unstained fine sand + coarse silt + POM (D), fine sand + fine silt + POM (E) and fine sand + clay + POM (F) mixtures are shown in the lower panel for comparison (image contrast was enhanced in this figure).
